# Outpatient psychiatric service utilization during the Covid-19 pandemic

**DOI:** 10.1186/s43045-022-00234-9

**Published:** 2022-10-03

**Authors:** Fateme Shirzad, Mohsen Shati, Seyede Salehe Mortazavi, Shakiba Gholamzad, Shahrzad Ahmadkaraji, Mahdie Pazhooyan, Narges Saeedi, Rana Hashemi, Saeedeh Shirdel, Mahdieh Salehi

**Affiliations:** 1 Department of Psychiatry, Spiritual Health Research Center, School of Medicine, Iran University of Medical Sciences, Tehran, Iran; 2 Mental Health Research Center, Psychosocial Health Research Institute, Iran University of Medical Sciences, Tehran, Iran; 3 Geriatric Mental Health Research Center, School of Behavioral Sciences and Mental Health (Tehran Institute of Psychiatry), Iran University of Medical Sciences, Tehran, Iran; 4 Student Research Committee, School of Behavioral Sciences and Mental Health (Tehran Institute of Psychiatry), Iran University of Medical Sciences, Tehran, Iran; 5 Mental Health Research Center, School of Behavioral Sciences and Mental Health (Tehran Institute of Psychiatry), Iran University of Medical Sciences, Tehran, Iran; 6 School of Behavioral Science and Mental Health (Tehran Institute of Psychiatry), Iran University of Medical Science, Tehran, Iran

**Keywords:** Covid-19, Psychiatric outpatient, Service utilization, Mental health, Abbreviation, OROdds ratio

## Abstract

**Background:**

The conditions related to the Covid-19 pandemic and quarantine have endangered the mental health of people in the community, especially psychiatric patients. This study aims to determine the mental health services usage of outpatient psychiatric patients who attended a public mental health clinic in Tehran, during the quarantine. The study sample consisted of 387 patients with an active record in the mental health clinic. They were selected using systematic random sampling. Data was gathered with the telephone interviews using the researcher-made checklist. The data were then collected and analyzed.

**Results:**

Participants in this study included 141 male and 264 female patients. One-hundred forty-two patients (36.7%) felt better during this period, and the rest got worse or did not make a difference. Among the patients who felt the need to visit, 144 (47.2%) referred for services, of which 81.7% had visited in person. Among the applicants, 84 (56%) succeeded in receiving the service. A total of 53.5% of patients had used at least one face to face, telephone, or online visit. Among them, women are compared to men (*p* = 0.002), educated people to low education (*p* < 0.001), and adults to children and the elderly (*p* = 0.02), and Tehran residents to foreigners (*p* = 0.01) used significantly more services.

**Conclusions:**

Experience with pandemic quarantine conditions has shown that face-to-face use of these services faces significant barriers. In this situation, the expansion of online services can help improve the condition of patients and should be on the agenda of mental health policymakers.

## Background

On March 11, 2020, the World Health Organization announced the new coronavirus as a global pandemic [1]. The world media and international organizations mostly emphasized the transmissibility and lethality of this virus [2]. It is argued that there is always a risk that coronavirus pandemic may lead to a secondary pandemic of mental health diseases in communities and health centers [3].

Delay in receiving psychiatric medications, lack of access to primary care or outpatient services, increased financial problems, being concern about being infected with coronavirus, staying at home for a long time, and poorer living conditions due to lack of resources in the weeks after the outbreak of the disease are factors contributing to the deterioration of mental health. These changes in circumstances may lead to feelings such as hopelessness and increased suicidal thoughts in patients with psychiatric problems [4].

People who had previous psychiatric conditions are more likely to be affected by the emotional state caused by the Covid-19 pandemic than the general population. It can lead to a worsening or recurrence of the illness [5]. They are more susceptible to be influenced by stressors and lack of regular access to mental health services. The lack of access is the result of quarantine restrictions, closure of many psychiatrists’ offices, difficulties to visit a psychiatrist or other mental health professionals, and the fear of getting sick at the time of attending to the psychiatrics’ office [4–6].

It is also important to strategically plan for services and resources to provide safe and high-quality psychiatric care as the number of people in need of care increases [4].

Better access to telemedicine services and hotlines plays important roles in minimizing the severity of psychiatric symptoms experienced by psychiatric patients [4].

Various types of online mental health services have been available to people in need during the pandemic in China. Online psychological interventions including cognitive-behavioral therapy for depression, anxiety, and insomnia have been developed [7]. However, integrated management policies and the national coordination of services are still insufficient. This can lead to unequal distribution and waste of medical resources and lack of evaluation of the efficiency of these services [8].

Predicting the prevalence of psychiatric disorders and becoming prepare for them for the future, and finding more knowledge about the needs of psychiatric patients in times of crisis, are essential [4]. However, there is no literature regarding the status of mental health delivery during the Covid-19 pandemic [9]. The data from this study can be used by mental health policymakers to design effective interventions and planning to eliminate insufficiency and provide desirable services to help the target group [10].

In Iran, with the rise of the corona from the end of 2020, the movement of citizens in the cities was limited, and for a period of several months, only medical center and centers related to the daily needs of the people were open. Psychiatric clinics were closed for a while, some services continued to be provided by telephone and online call and chats, and in the inpatient wards, we only admitted patients whose hospitalization was mandatory, and the capacity of the wards was also limited. We did not have teleservices in public hospitals. Teleservice was provided in a limited way in some private clinics, and we did not have it in public hospitals.

The purpose of the present study was to investigate the status of patients with psychiatric disorders’ usage of mental healthcare services.

## Methods

### Design and sample

This study is a cross-sectional study that was conducted to determine the status of utilization of mental health services of outpatient psychiatric patients attending the clinic of the Faculty of Behavioral Sciences and Mental Health, Tehran, Iran, during the Covid-19 pandemic and quarantine. The clinic is a psychological-psychotherapy clinic that is not connected to the hospital; for this reason, most of its patients are patients with mild psychiatric disorders of lower severity who need psychological-psychotherapy services, and severe psychiatric patients are less likely to visit this center.

The statistical population of the study was all patients who attended this clinic during the 6 months before the start of Covid-19.) Due to the fact that the outbreak of the covid disease in Iran was officially announced on 30th of February 2020, the patients who came to the center from the beginning of September were included as a statistical population.) In the mentioned period, 1018 clients have attended the clinic. Among them, 387 patients’ records were selected by systematic random sampling.

### Procedure

Data was collected using a researcher-made checklist. The research team who was involved in the checklist designing were 3 psychiatrists, 5 psychologists, and a methodologist. It was then handed out to 3 other psychiatrists to ensure the comprehensiveness and validity of its contents. The checklist was also assessed and underwent final approval by the same 3 psychiatrists in terms of text understandably and face validity.

The checklist items were deterioration or improvement of the symptoms during the period, the dosage of medications, reasons for not taking the medications or taking them, and the extent to which they feel needing services during, the extent to which the services are used or the reasons why such measures are not taken despite feeling the need for them, and the level of satisfaction with the abovementioned services.

The study protocol was approved by University Research Ethics Committee. The patient’s contact details, their demographic information (age, gender, education, employment, residence, marital status), and the diagnosis of the patients were extracted from their medical records. The patients were then contacted by the psychologists from the research team, and their checklist data were completed. The records with missing contact or demographic information or ones without diagnoses were excluded. Patients were interviewed by telephone. Each phone call lasts between 7 to 10 min. The questions were asked from their informed caretakers if the patients were unable to answer independently (children or elderly).

### Statistical analyses

After data description using appropriate descriptive statistics, we use the independent *t*-test, one-way ANOVA, and χ^2^ test to measure differences in distribution and prevalence of study variables across different subgroups. Also, we used binary logistic regression to do multivariate analysis and examine associations between target variables. We reported adjusted odds ratios (ORs) with 95% CIs. We did all analyses with PASW Statistics (version 21).

### Ethical considerations

This study is approved by the ethical committee of the Iran University of Medical Sciences (IR.IUMS.REC.1399.471). The purpose of the research was explained to all participants before each interview; the patient was informed about the procedure of the research. Their willingness to participate in the study was verbally asked. They were assured about the confidentiality of their information would and the anonymous publication of results. They were also assured that their refusal to participate in the study would not affect their therapy. Then, inform consent was obtained orally from them.

## Results

Three-hundred eighty-seven patients participated in the present study. The detailed demographic information is presented in Table 1.

**Table 1:** Demographic characteristics of study sample

Variables		Frequency	Percent
Gender	Male	141.0	36.4
	Female	246.0	63.6
Age	0–18	91.0	23.8
	19–59	252.0	66.0
	60+	39.0	10.2
Education	High school	121.0	35.5
	Diploma	109.0	32.0
	Academic	111.0	32.5
Marital status	Single	189.0	50.1
	Married	178.0	47.2
	Divorced	10.0	2.7
Residency	Tehran	345.0	90.1
	Other city of Iran	38.0	9.9
Job	Unemployed	175.0	45.2
	Employed	103.0	26.6
	Housewife	109.0	28.2
Psychiatric disorder	Anxiety and OCD	158.0	40.8
	Mood disorder	117.0	30.2
	ADHD	64.0	16.5
	Others	48.0	12.5

In this study, in 144 patients (47.2%) of those who felt the need to apply for some kind of service, 81.7% of them applied for in-person service, and the rest applied for the service by phone or online visit. Only 56% of people who applied for in-person service were able to receive the service. A total of 85/2% of the services are provided by a psychiatrist, 11.3% by a psychologist, and the rest by both jointly and as a team. The average number of fog service receipts was 2.14, and the standard deviation was 1.7.

The patients evaluated their illness conditions in this period as follows: 142 patients (36.7%) felt that they were recovered in this period compared to the past, while 83 patients (21.4%) felt that their symptoms have become worse in this period, and the rest of them had an unchanged condition.

Based on Table 2, the change in the illness conditions during the past 3 months was significantly different between the two genders (*p* < .001). It was often unchanged in males (54.6%), while females mostly felt they have improved compared to the past (42.7%). The level of education was another effective factor (*p* = .04). Half of the patients with pre-diploma or diploma level education stated that their illness conditions had not changed compared to the past (48.8% and 42.2%), while a larger percentage of those with higher education felt their illness conditions were improved (48.6%).

**Table 2:** Change in disease status during the epidemic in terms of demographic and contextual variables

Variable		Unchanged		Worse		Better		*p*-value
		*N*	%	*N*	%	*N*	%	
Gender	Male	77	54.6	27	19.1	37	26.2	< .001
	Female	85	34.6	56	22.8	105	42.7	
Age	0–18	47	51.6	15	16.5	29	31.9	0.21
	19–59	95	37.7	58	23.0	99	39.3	
	60+	18	46.2	7	17.9	14	35.9	
Education	High school	59	48.8	25	20.7%	37	30.6	.043
	Diploma	46	42.1	25	22.9%	38	34.9	
	Academic	35	31.5	22	19.8%	54	48.6	
Marital status	Single	90	47.6	38	20.1%	61	32.3	.059
	Married	64	36.0	43	24.2%	71	39.9	
	Divorced	4	40.0	0	0	6	60	
Job	Unemployed	85	48.6	38	21.7%	52	29.7	.034
	Employed	39	37.9	17	16.5%	47	45.6	
	Housewife	38	34.9	28	25.7%	43	39.4	
Residency	Tehran	146	42.3	69	20.0%	130	37.7	0.128
	Other city of Iran	13	34.2%	13	34.2%	12	31.6%	
Psychiatric disorder	Anxiety and OCD	70	44.3%	34	21.5%	54	34.2%	0.625
	ADHD	41	35.0%	26	22.2%	50	42.7%	
	Mood disorder	28	43.8%	15	23.4%	21	32.8%	
	Other	23	47.9%	8	16.7%M	17	35.4%	

Employment status was also effective in changing the patients’ illness condition (*p* = .03). About half of unemployed patients felt no change in their illness conditions (48.6%), while employed patients reported a better illness condition compared to the past (45.6%). Age (*p* = 0.21), marital status (*p* = .05), the type of disorder (*p* = 0.62), and place of residence (*p* = 0.12) were not significantly associated with changes in the illness condition during the pandemic.

There was no significant relationship between taking the drugs according to the prescriptions and the diagnosis (*p* = 0.28). There was also no significant relationship between the feel for using mental health services and the diagnosis (*p* = 0.95). There was no significant difference between males and females in the feeling of need for using services (*p* = 0.11).

A binary logistic regression was used to determine the factors associated with users of mental care services. Participants who had used services by phone, online, and in-person were categorized as the service users’ group, and the rest of the patients fell into the non-user group. Among participants who announced that they needed services, 46.5% did not use any type of services. Among them, 53.5% had used to at least one type of service. According to Table 3, gender, education, age, and place of residence were significantly associated with the patients’ usage of the services. Females used service more compared to males. The use of services varied according to the level of education of the individuals, and the participants with academic education used the services more. Children and the elderly had less use of the services than participants with age 19–59. Patients living in Tehran had more use for the services than patients who were living in other cities.

**Table 3:** Status of service use based on demographic variables

Variable		Non-user group		user group		*P*-value
		Number	%	Number	%	
Gender	Male	61	58.7	43	41.3	.002
	Female	78	40.0	117	60.0	
Education	High school	49	55.7	39	44.3	< .001
	Diploma	41	51.2	39	48.8	
	Academic	26	28.3	66	71.7	
Age	Less than 18	36	58.1	26	41.9	.02
	19–59	81	40.1	121	59.9	
	More than 60	17	56.7	13	43.3	
Marital status	Single	70	49.3	72	50.7	0.47
	Married	60	42.6	81	57.4	
	Divorced	5	55.6	4	44.4	
Residency	Tehran	114	43.5	148	56.5	.01
	Other city of Iran	22	66.7	11	33.3	
Job	Unemployed	67	50.0	67	50.0	0.38
	Employed	35	47.3	39	52.7	
	Housewife	37	40.7	54	59.3	
Psychiatric disorder	Anxiety and OCD	59	42.4	63	39.4	0.51
	ADHD	25	18.0	23	14.4	
	Mood disorder	37	26.6	55	34.4	
	Other	18	12.9	19	11.9	
Adherence	Yes	88	68.2	100	69.2	0.34
	No	41	31.8	59	37.1	

In the multivariate analysis, variables with a significant association with usage of the mental care services that were found in the bivariate analysis were included in the logistical regression model. The final results of the model are shown in Table 4. Females had the chance to use the services twice as much as males. The higher level of education had also a significant association with the use of the services, and patients not living in Tehran had 64% less use of the services compared to those who were living in Tehran. There was no significant statistical association between age and use of the services in the multivariate analysis.

**Table 4:** Logistic regression analysis of service utilization associates

	Beta	*p*-value	Adjusted OR	*CI*_95%_ for adjusted OR	
Gender	0.774	0.007	2.168	1.237	3.802
Age	−0.237	0.343	0.789	0.484	1.288
Education	0.624	< 0.001	1.867	1.321	2.639
Residency	−1.010	0.018	0.363	0.156	0.843

Also, about one-third of patients (32.3%) did not do that, and no medicine was prescribed for 39 patients (10.4%).

The following are the reasons for not taking the medicines: feel no need to take the medicines (16%), the pharmaceutical side effects (33.6%), running out of the medicines and had no access to a therapist for a visit and renewal of the prescription (27.5%), being disappointed about the effectiveness of the drugs (11.5%), and other reasons (11.4%).

**Fig 1:**
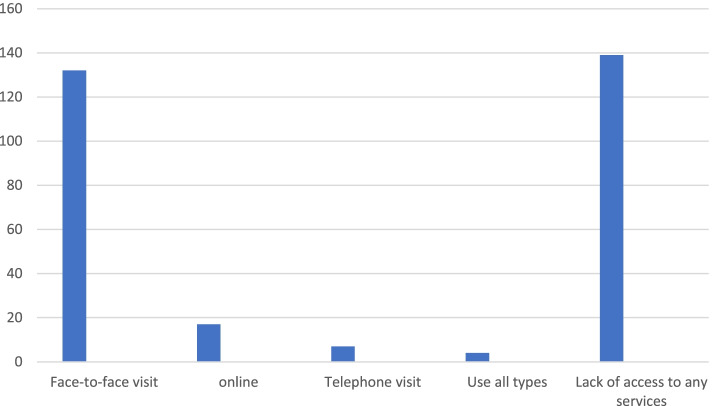
Patients’ access to each service during quarantine

Regarding the feeling of the need for a mental care visit or other services, 294 patients stated that they felt the need to visit their psychiatrist or psychologist during this period, and 92 patients said that they felt no need. As shown in Fig. 1, among the people who felt the need to access psychological services, the highest access was face-to-face visit, followed by online visit and finally telephone counselling.

The chief complaints of 44 patients (34.6%) were relieved to a great extent and moderately for 61 patients (41%). According to the results, there was no significant difference in the extent to which the problems were solved following the use of healthcare services among the patients with different diagnoses (*p* = 0.74).

## Discussion

We discuss the results in two separate sections: changes in the patients’ condition and factors affecting their utilization of services.

### Changes in the patients’ condition

According to our findings, the level of recovery was more in women, employed, and patients with higher education than men, unemployed, and those with a lower level of education. This finding is not unexpected, and it is consistent with previous literature regarding factors influencing the prognosis in psychiatric disorders [11].

Our study showed that the majority of participants who attended psychotherapy during this period were women, and the most frequent diagnosis was anxiety and obsessive-compulsive disorders. This is in line with previous knowledge in Iran that anxiety and depression are the two most common psychiatric disorders, and that they are more common in women than men [12]. Our results were also consistent with the findings of a previous study that showed that the Covid-19 pandemic caused more worry about cleanliness, excessive monitoring of physical fears, and too much fear of infection, which could intensify anxiety and obsession in people [13].

Regarding the rate of medication use in psychiatric patients, previous studies have shown that adherence to treatment in patients is generally about 50% [14]. In our study, 50% of patients used their medication based on their doctor’s prescription. The reasons for nonadherence to treatment were as follows: (1) running out of drugs, (2) drug side effects, and (3) becoming disappointed with the improvement of the disease by using the drugs. These are the three factors that were among the main causes of psychiatric patients’ nonadherence to treatment, even before the Covid-19 pandemic [15]. The important point is that all of these three factors can be minimized by increasing access to physicians and mental health services.

### Factors affecting the patient’s level utilization of services

Our results showed that self-assessment of health status is an important factor in requesting mental health services. Patients who had a bad evaluation of their disease used more services. Moreover, some demographic factors such as gender, age, and f residence have played important and precipitating roles in the accessibility of services. Women used mental health services twice as much as men. It may be associated with a greater feeling of need for seeking help in women with psychiatric illness than men [16]. This finding is not specific to the coronavirus era and has been found in a previous study conducted in Iran [17]. Age was also related to the higher-level usage of the service. Children and the elderly, who are more dependent on their families to attend mental health centers and have less ability and independence to use online services, had less access to the health services. However, this difference was not statistically significant. The higher education level (college degree) was related to the increase in the level of usage of the service, and on the other hand, it was related to a better disease condition. It should be noted that higher education itself increases the chance of employment, and the education level and employment status increase each other’s role by creating a more stable social network and more effective social performance. Perhaps, the role of higher education and employment cannot be interpreted separately [18].

Place of residence also played a precipitating role in the use of mental health services during quarantine. People living in Tehran had significantly more access of mental health services. This factor can be justified both by urban facilities and easier transportation in Tehran as the capital and by the ban on cross-city trips during the quarantine for patients not residing in Tehran which caused them some limitations. Furthermore, the fear of becoming infected with the coronavirus during the travel to clinics can be considered as one of the reasons why residents of Tehran used the services more than nonresidents. In our study, this factor was the concern of 60% of the patients who felt the need for a psychiatric intervention, but they did not visit a therapist. In a situation like this, using online services can be a solution. Unfortunately, there are plenty of barriers in the use of these methods, such as how to keep patient information confidentiality (especially in online methods), therapists not being familiar with the use of such technologies in treatment, and the lack of high-quality Internet infrastructure — which reduced patients’ access to these services. These were among the factors that had reduced the usage of such services, especially in the early days of the quarantine in Iran [19].

Patients with no insurance who had to pay for all health services and had poorer social resources used these services less than normal. In particular, the insurance companies did not cover mental health services that were provided by phone or online, which leads to a higher cost of treatment; it was one of the most important obstacles leading to a lower level of access in our study.

In our study, about 50% of patients did not have used any type of mental health service. Even available services were not properly advertised. Therefore, some patients stated that they were not aware of mental health clinic services being active during the quarantine. However, such problems are not limited to our country. The latest World Health Organization report stated that in 130 countries, mental health services are severely damaged during the corona pandemic and need immediate attention [20]. The provision of mental health services by policymakers was not a priority [21]. Patients with psychiatric disorders, during this period, were more exposed to psychological problems due to reasons such as the diminished activity of their social networks, reduced support of friends and family members, unemployment and its related economic crisis, and lack of insurance [22].

Besides the need that policymakers have to pay more attention to mental health services, psycho-educators, and the society about the possibility of psychological trauma in this period, the personal and family resources of patients should be strengthened by providing awareness and creating motivation in the society, patients, and their families. It is an effective factor in their use of services. Therefore, providing counselling services and psychoeducation is an enabling factor for people to use mental health services. Also, psychoeducation itself is an effective mental health service. Our study showed that psychoeducation can be very helpful in any form (in-person, online, or even for a short time). In our study, more than 80% of patients, regardless of their disorder, expressed that by using counselling services, their psychological problem was improved.

Finally, we can discuss the findings of this study with the Anderson model which is a model that was used in previous studies to examine the factors affecting access to mental health services [23]. Demographics such as gender, age, place of residence, and level of education were significant factors that were significantly associated with the use of services. The lack of social support resources such as insurance coverage for online and telephone services was among the reasons that caused patients to use these services less frequently. In our study, the most important variable expressed in this model (need factor) was the participant’s assessment of their mental health, which was related to using of services.

### Limitations

This study was carried out in a university-affiliated clinic in Tehran with relatively large sample size. There is a need for a multicenter study with a larger sample size to generalize the results with more precision.

## Conclusions

During Corona quarantine, problems with using face-to-face mental health services led to the proliferation of online and offline services. Despite important limitations in this type of services, such as high-speed Internet access and problems related to patients and therapists not being familiar with its mechanism, but in times of crisis, it provides cheaper and faster access to more patients. Therefore, preparing and expanding the platform of these services can enable us to deal with such crises in the future.

## Data Availability

Not applicable
